# Supplementary figures and images for: CD44 as a novel therapeutic target in pulmonary arterial hypertension: Insights from multi-omics integration and molecular docking
Source: PLoS One. 2025 Sep 19;20(9):e0332817. doi: 10.1371/journal.pone.0332817 (PMC12449029; doi:10.1371/journal.pone.0332817)

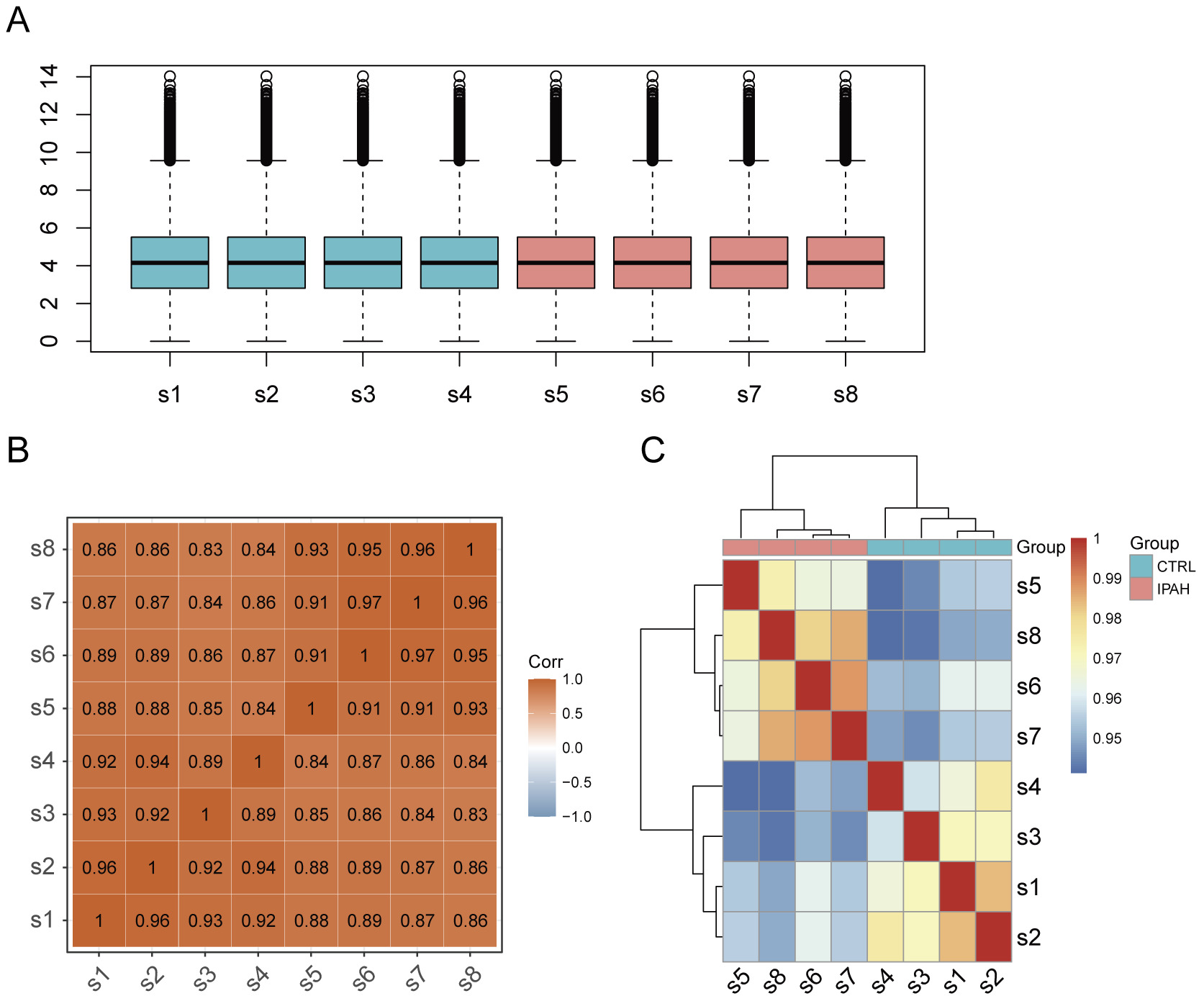

Supplement: S1 Fig — A: Boxplots showing gene expression distributions across all samples. B: Heatmap of Pearson correlation coefficients between all samples. C: Hierarchical clustering of samples based on transcriptomic similarity, with CTRL samples in blue and IPAH samples in red. (TIF) [file pone.0332817.s001.tif]

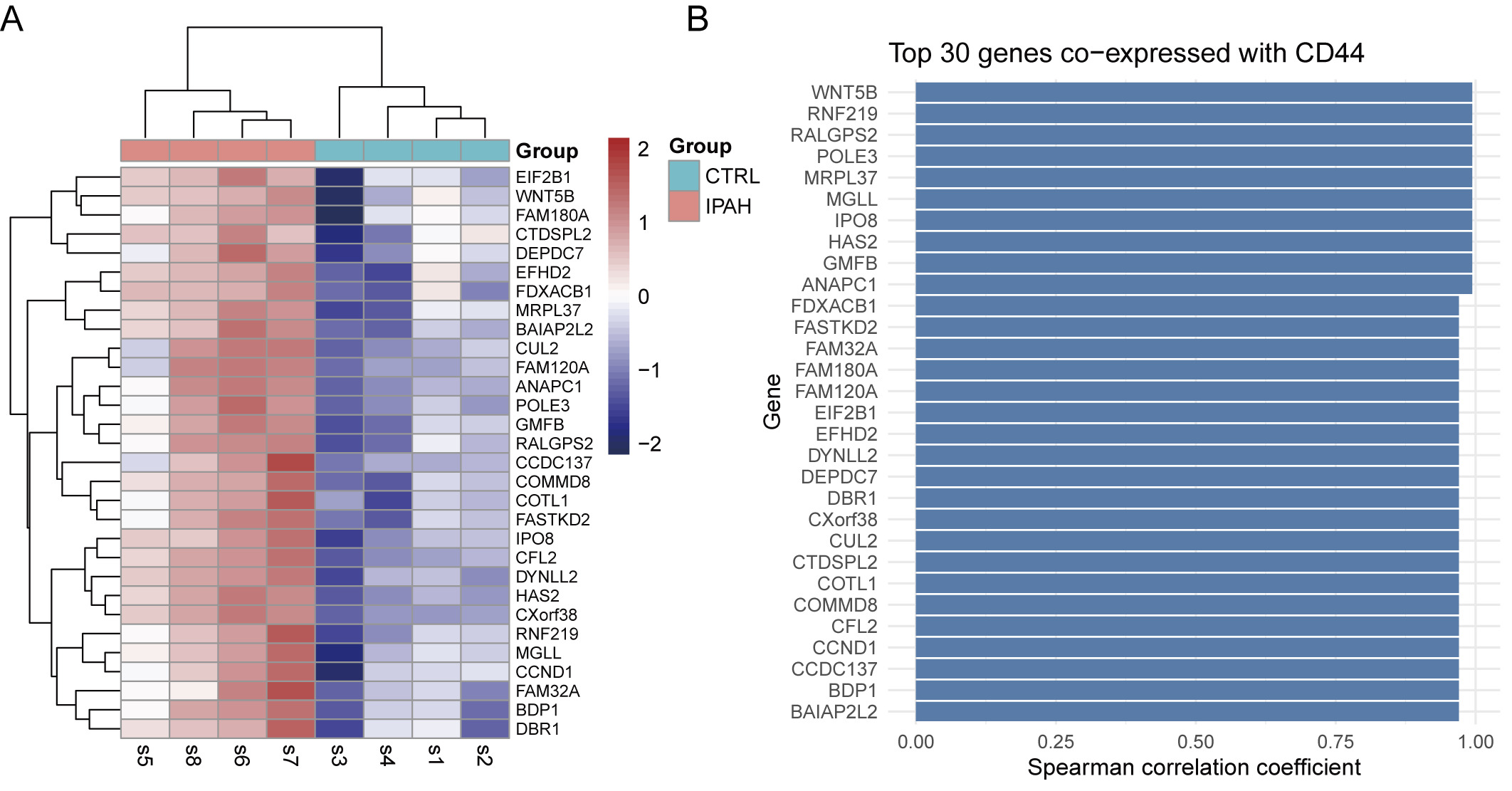

Supplement: S2 Fig — A: Heatmap showing expression patterns of the top 30 genes most strongly co-expressed with CD44 based on Spearman correlation in bulk RNA-seq data from IPAH and control samples. B: Bar plot ranking the top 30 genes by absolute Spearman correlation coefficient. (TIF) [file pone.0332817.s002.tif]

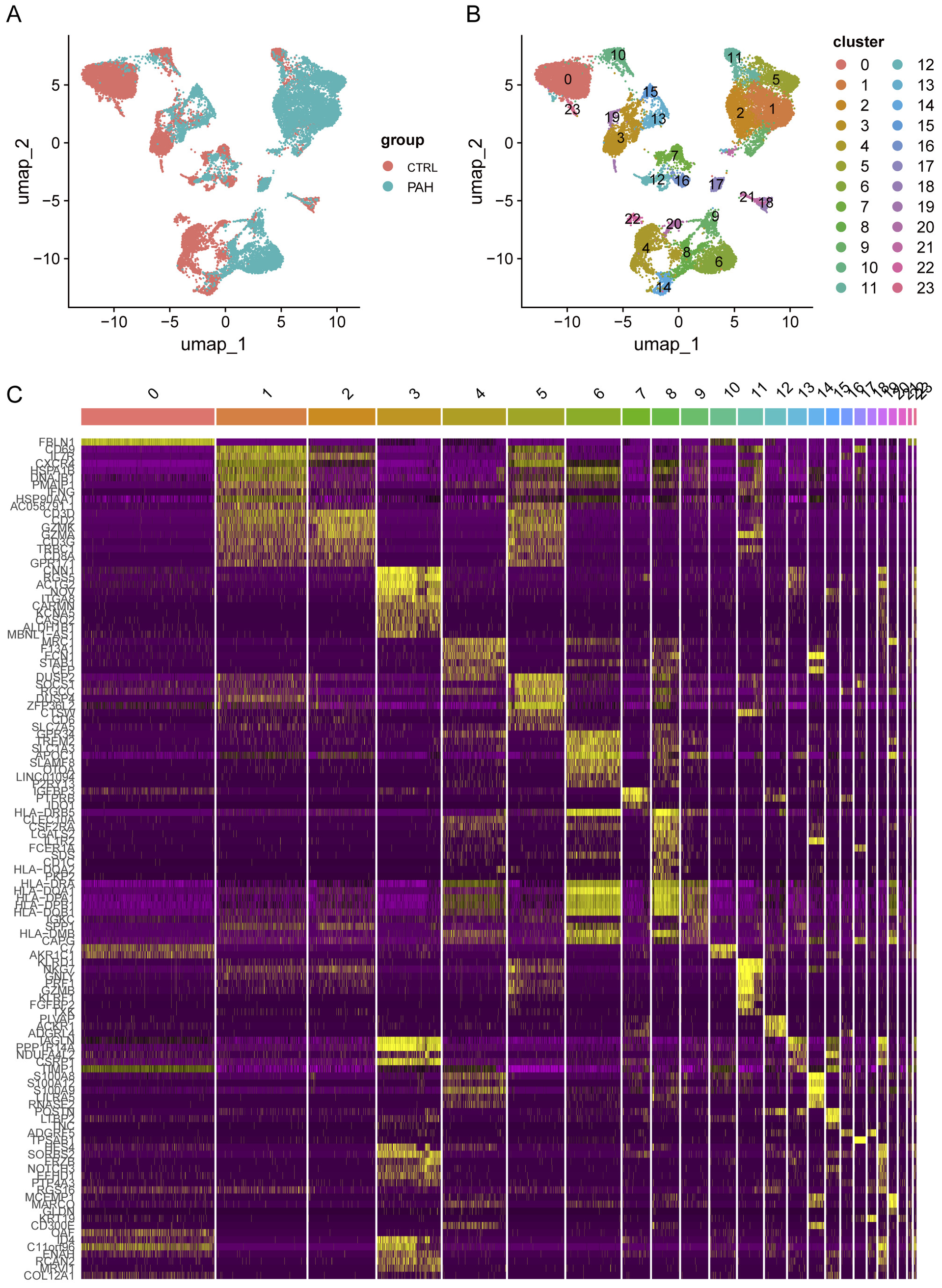

Supplement: S3 Fig — A: UMAP plot showing integration of cells from CTRL and IPAH groups, colored by condition. B: UMAP plot displaying 24 identified clusters, each labeled with its cluster number. C: Heatmap of representative marker genes across clusters. (TIF) [file pone.0332817.s003.tif]

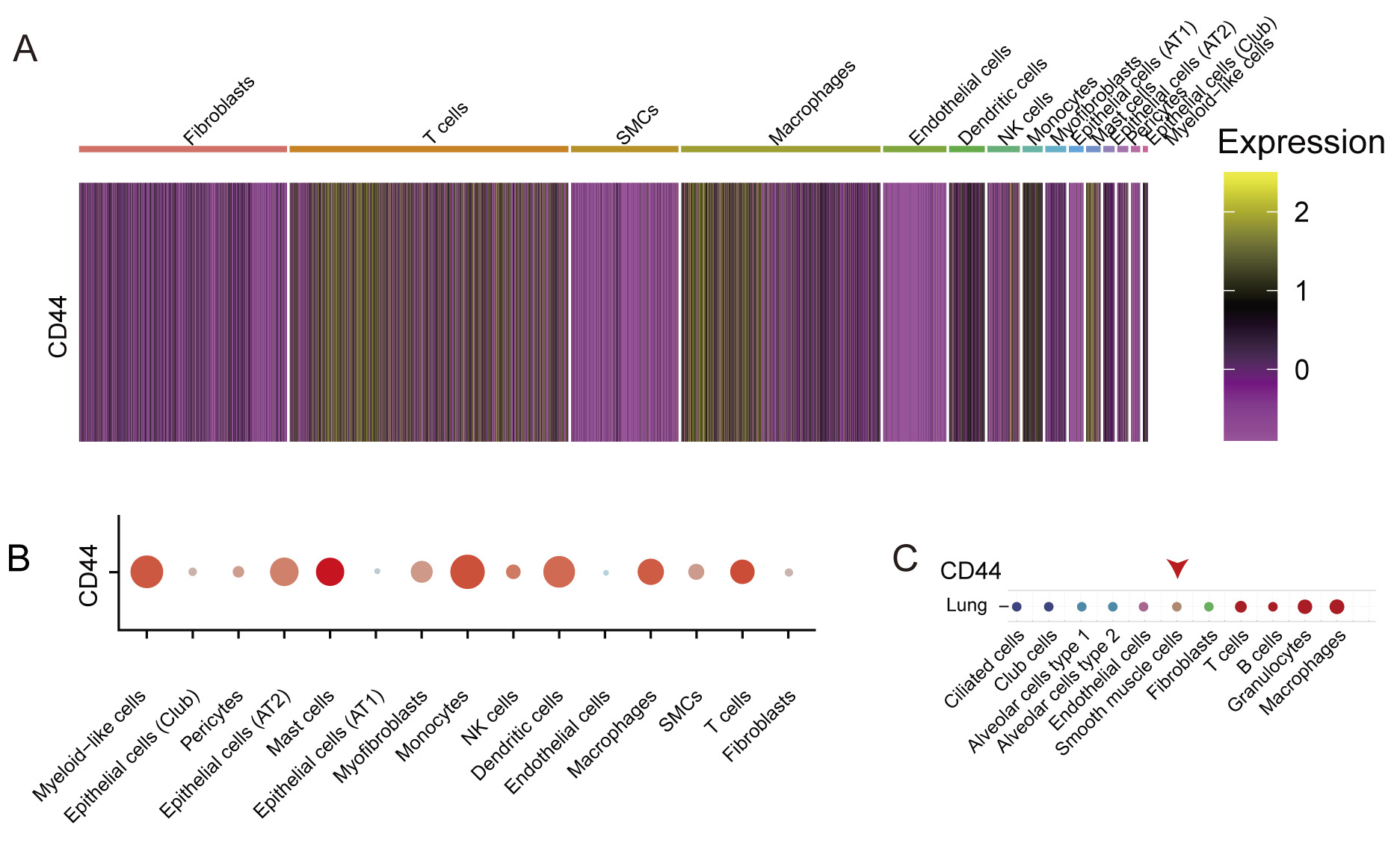

Supplement: S4 Fig — A: Schematic representation of CD44 expression across annotated cell types. B: Dot plot analysis of CD44 expression across distinct cell types in this lung scRNA-seq data. C: Validation of CD44 protein expression across distinct cell types in human lung tissue from the HPA database. (TIF) [file pone.0332817.s004.tif]

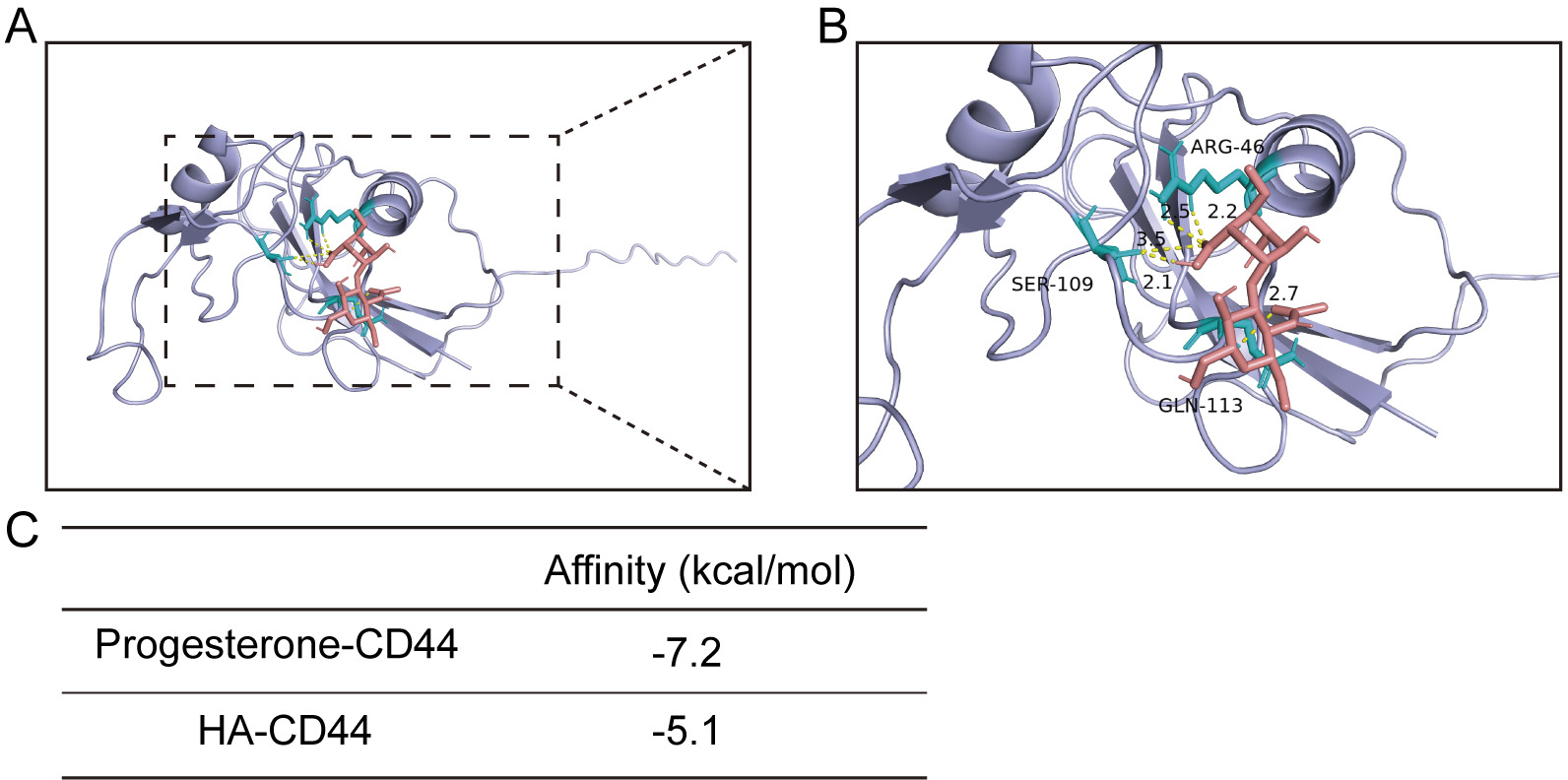

Supplement: S5 Fig — A: Docking pose of HA binding to CD44. B: Hydrogen bond interactions between HA and key residues of CD44, including ARG-46, SER-109, and GLN-113. C Predicted binding affinities of progesterone and the natural ligand HA with CD44. (TIF) [file pone.0332817.s005.tif]
