# Supplementary material for: CD44 as a novel therapeutic target in pulmonary arterial hypertension: Insights from multi-omics integration and molecular docking
Source: PLoS One. 2025 Sep 19;20(9):e0332817. doi: 10.1371/journal.pone.0332817 (PMC12449029; doi:10.1371/journal.pone.0332817)
Supplement: Attachment — Submitted filename: Comments_R1.pdf [file pone.0332817.s009.pdf]

Comments

The manuscript titled **"CD44 as a Novel Therapeutic Target in Pulmonary Arterial Hypertension: Insights from Multi-Omics Integration and Molecular Docking"** outlines research identifying **CD44 as a potential therapeutic target for pulmonary arterial hypertension (PAH)**, a severe and often fatal lung disorder. Through the analysis of both bulk and single-cell RNA sequencing data, the authors found that **CD44 expression is elevated in PAH patients**, particularly in T cells, suggesting its involvement in the inflammatory processes that drive the disease. Further computational analysis, specifically molecular docking, indicates that **progesterone could potentially interact with CD44**, offering a possible pathway for therapeutic intervention to reduce inflammation and improve patient outcomes.

Although manuscript follow a multilayered approach to answer the role inflammation in PAH, still it raises following question.

1. The introduction lacks a critical discussion on prior CD44 studies in PAH (Ref 15 could be elaborated)
2. The bulk RNA-seq analysis was conducted on samples from only 4 iPAH patients and 4 healthy controls. Similarly, the single-cell RNA-seq analysis used data from 3 iPAH patients and 3 controls. These are relatively small sample sizes, which can limit the statistical power of the study and the generalizability of the findings to the broader PAH patient population. This should acknowledged as a limitation.
3. Add **supplementary files** with complete DEG lists and enriched pathway tables.
4. The Materials and Methods section and the Data Availability statement consistently refer to the single-cell RNA-seq dataset as GSE228644. However, the legend for Figure 3, which presents the scRNA-seq results, states the data is from GSE231993. This is a clear inconsistency that needs to be corrected.
5. Enrichment tools and thresholds are standard, but exact GO/KEGG terms and p-values should be added in at least in Supplementary Data.
6. PPI networks predict interactions but do not confirm functional effects experimentally. Lacks complementary **co-expression analysis (from RNA-seq data)** to strengthen findings.
7. The molecular docking results suggesting a high-affinity interaction between progesterone and CD44 are based on computational predictions. While this provides a novel hypothesis for therapeutic intervention, these findings are preliminary and require experimental validation (e.g., in vitro binding assays, cellular or animal model studies) to confirm the interaction and its

functional significance in a biological context.

8. Molecular docking analysis seems overexerted as the docking energy (−7.2 kcal/mol) is reasonable but not definitive. Moreover, lacks comparative analysis with known CD44 ligands (e.g., HA analogs) and no molecular dynamics simulation was performed to support binding stability. Therefore looking at aforesaid limitations, the identification of progesterone as a CD44 ligand is speculative, thus needs a more cautious tone or follow-up validation. Statements like "These results position progesterone as a potential CD44 antagonist" are premature without functional assays.
9. Statement: "highlighting systemic immune suppression in advanced PAH" contradicts the theme that inflammation is increased and requires further clarification.
10. Author should discuss about unintended consequences of CD44 inhibition.

Minor

- Fix **typographical and grammatical errors** throughout.
- Improve **figure quality and annotations**.
